# Biochemical characterization of a novel purified lectin extracted from *Pleurotus ostreatus* mushroom for its antiviral activity

**DOI:** 10.1038/s41598-025-09967-z

**Published:** 2025-07-31

**Authors:** Yousra A. El-Maradny, Marwa M. Abu-Serie, Mona H. Hashish, Heba S. Selim, Abdulrahman M. Saleh, Esmail M. El-Fakharany

**Affiliations:** 1 Protein Research Department, Genetic Engineering and Biotechnology Research Institute, City of Scientific Research and Technological Applications (SRTA-City), New Borg AL-Arab, Alexandria, 21934 Egypt; 2 Department of Medical Biotechnology, Genetic Engineering and Biotechnology Research Institute, City of Scientific Research and Technological Applications (SRTA-City), New Borg AL-Arab, Alexandria, Egypt; 3 Microbiology Department, High Institute of Public Health, Alexandria University, Alexandria, Egypt; 4 Department of Pharmaceutical Chemistry, Faculty of Pharmacy, Cairo University, Kasr El-Aini Street, Cairo, 11562 Egypt; 5 Pharmaceutical and Fermentation Industries Development Centre (PFIDC), City of Scientific Research and Technological Applications (SRTA-City), New Borg Al-Arab, Alexandria, Egypt; 6 Pharos University in Alexandria, Canal El Mahmoudia Street, Beside Green Plaza Complex, Alexandria, 21648 Egypt

**Keywords:** Mushrooms, Lectins, Antiviral, HCV, HBV, Biochemistry, Biotechnology, Microbiology, Pathogenesis

## Abstract

Viruses are responsible for numerous serious outbreaks and pandemics worldwide. In this context, lectins, which are carbohydrate-binding proteins, have garnered attention due to their antiviral properties against RNA and DNA viruses. The antiviral potential of the purified and well-characterized lectin from *Pleurotus ostreatus* (POL) was assessed against various viruses. POL showed potent antiviral activity against HCV with IC_50_ values of 68.75 nM and 52.13 nM for determining the blocking and neutralizing infectivity, respectively. POL exhibited IC_50_ values of 42.75 and 14.88 nM against HBV through treatment and blocking mechanisms, respectively. Notably, POL demonstrated a 58.80% binding capacity to cell-surface CD81, while the IC_50_ values for targeting the scavenger receptor class B-type I (SR-B1), HCV-NS3/4A protease inhibition, and anti-HBV polymerase activity were established at 10.08, 10.98, and 4.22 nM, respectively. The outcomes of this investigation offer crucial insights into the mechanisms through which POL effectively inhibits infection caused by different viruses. These findings have the potential to inform the development of antiviral strategies and therapeutic interventions.

**Supplementary Information:**

The online version contains supplementary material available at 10.1038/s41598-025-09967-z.

## Introduction

Viruses, particularly RNA viruses, cause many serious outbreaks and pandemics around the world, resulting in high mortality rates and economic problems^1^. According to the World Health Organization (WHO), 58 and 296 million people are infected with chronic hepatitis C virus (HCV) and hepatitis B virus (HBV) infections, respectively. Viral hepatitis (VH) is responsible for almost one million fatalities worldwide, with HBV and HCV accounting for 820,000 and 290,000 deaths, respectively^2,3^. Despite the availability of an active and effective HBV vaccine, all available therapies to treat HBV are not able to completely remove the virus or eradicate the integrated covalently closed circular DNA (cccDNA), but rather inhibit viral replication^4,5^. The new treatment for HCV using direct acting antivirals (DAAs) is very effective and targets three proteins: non-structural (NS) NS3/NS4A protease, NS5A region, and NS5B polymerase^6^. However, the high cost of these drugs remains a key barrier to their widespread use^7^.

The most challenging part of selecting antiviral medicines is determining an effective and safe antiviral drug that does not destroy host cells^8^. Furthermore, the advent of untreatable illnesses and microorganism resistance to available antimicrobial medications enhance the demand for novel drugs. Antiviral screening of natural compounds and their derivatives is a promising source^9,10^. These sources are rich in bioactive chemicals with unique chemical structures that might be used to develop new antiviral drugs^11^. Mushrooms are a rich source of proteins, glucans, vitamins, ergosterol and important minerals with anti-tumor, antiproliferative, and immunomodulatory activity^12,13^. Lectins are non-immunoglobulin glycoproteins that are capable of binding to a wide range of sugar molecules with high selectivity and stereo specificity without changing the ligands of glycosyl^14^. Lectins have a role in cellular signaling, cancer, glycoprotein scavenging from the circulatory system, cell–cell interaction in the immune cells, differentiation, and protein binding to cellular components, as well as host defense, inflammation, and metastatic mechanisms^15^. Mushroom lectins have gotten a lot of attention recently because of their anti-proliferative, antibacterial, and immunomodulatory characteristics^16–18^. The aim of this study was to purify and characterize a lectin from *Pleurotus ostreatus* (POL), evaluate its in vitro antiviral activity against HBV and HCV, and investigate its potential mechanisms of action. The result can emphasize the usefulness of the mushrooms and its lectin content as a natural source of antiviral compounds and may open a window to discover alternative agents to substitute the current antiviral products and finding novel compounds that can fight the emerged viral diseases.

## Results

### Mushroom lectin (POL) purification

**Fig. 1:**
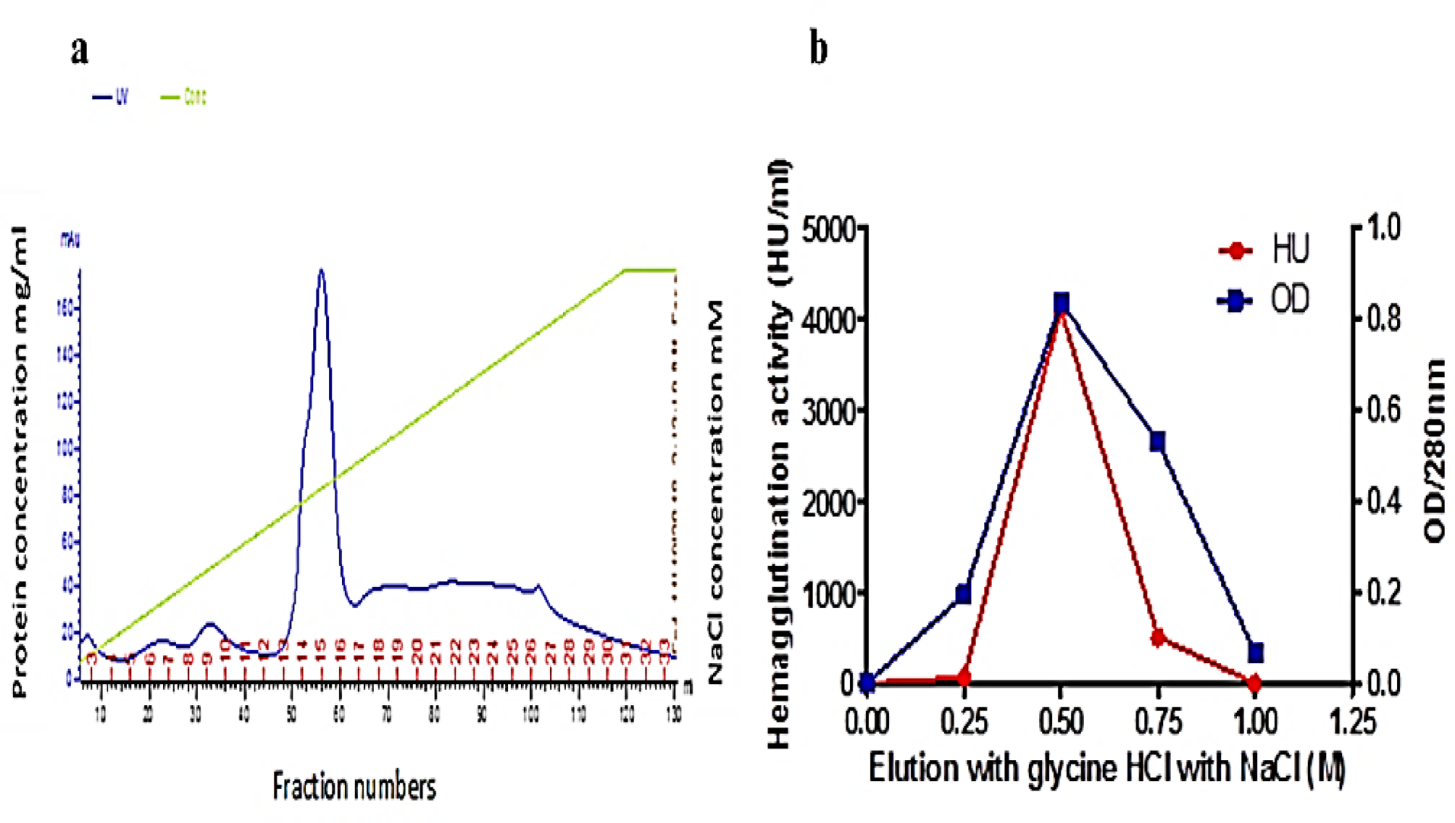
(**a**) A typical FPLC elution profile for the chromatography of POL on mono-Q column previously equilibrated with phosphate buffer (pH = 7.4), eluted with the same buffer containing 1 M NaCl. The elution was at flow rate 3 mL/fraction and absorbance measured at 280 nm. (**b**) Fetuin affinity purification of POL.

**Fig. 2:**
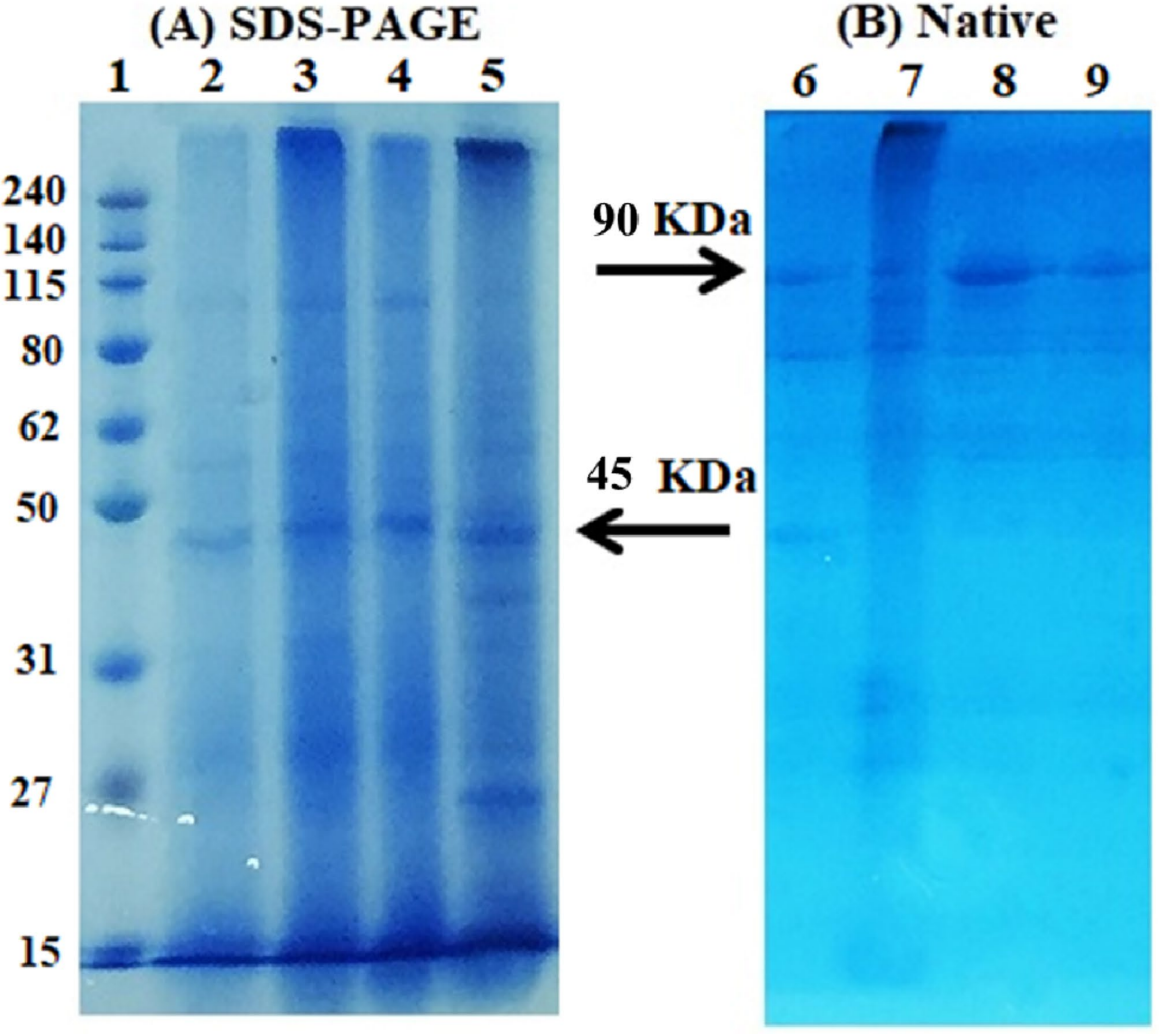
Protein gel electrophoresis of *P. ostreatus* mushroom using (**A**) 15% SDS PAGE: lane 1; protein ladder, lane 2; eluted protein from fetuin column, lane 3; eluted protein from mono Q column, lane 4; 60% (NH_4_)_2_SO_4_ precipitate, lane 5; crude extract, and (**B**) Native gel electrophoresis: lane 6; eluted protein from mono Q column, lane 7; crude extract, lanes 8 and 9; eluted protein from fetuin column.

The elution profiles obtained from the mono-Q column using FPLC for the crude extract are depicted in Fig. 1a. The fractions displaying a notable protein concentration (14, 15, and 16) were collected. To assess their lectin content, a hemagglutination (HA) test was performed, and subsequently, these fractions were loaded onto the fetuin affinity column. Figure 1b; Table 1 showcase the results of the fetuin column elution and purification steps of lectin, respectively. The elution performed with 0.5 M NaCl demonstrated the highest concentration at 280 nm, measuring 0.835, along with an HA titer of 4096 units of lectins. This was followed by an elution utilizing 0.75 M NaCl. The molecular weight of the purified lectin was determined through SDS-PAGE (Fig. 2A), illustrating the progression of the purification steps for *P. ostreatus*. In this regard, lane “2” distinctly displayed the purified POL, characterized by a singular band with an approximate molecular weight of 45 kDa. Additionally, an analysis using native gel electrophoresis with the purified POL (depicted in Fig. 2B) revealed the presence of a solitary band, indicating a molecular weight of approximately 90 kDa. This comparison underscores the variations in molecular weight based on the separation technique employed.

**Table 1:** Purification scheme of POL shows that using ammonium sulphate at final concentration 60%, resulting in POL specific activity of 97.52 u/mg, and 41.63 purification fold increases in purity.

Purification steps	Volume (mL)	Protein concentration (mg/mL)	Total protein (mg)	HA (Titer)^a^	Total activity (U)	Specific activity(U/mg)	Yield/ recovery^b^	Purification level
Crude	500	218.54	109268.9	512	256,000	2.34	100	1
60% Amm. sulfate	100	164.40	16440.38	2048	204,800	12.46	80	5.32
Mono Q elution	25	109.05	2726.284	1024	25,600	9.39	10	4.01
**Fetuin elution**	15	42	630	4096	61,440	97.52	24	41.63

^a^HA: hemagglutination activity. The reciprocal of the end-point dilution demonstrating hemagglutination with human erythrocytes was designated as titer.

^b^Yield/Recovery of lectin activity was based on the crude extract.

### Characterization of the purified lectin (POL) from mushroom

Table 2 illustrates the hemagglutination (HA) activity of the purified POL. It is noteworthy that the highest titers were observed, measuring 4096 HU/mL for the human group O and 256 HU/mL for the AB group.

**Table 2:** Hemagglutination units of the purified POL.

Purified lectin (POL)	Hemagglutination unit (HU/mL)				
	Rabbit	AB	A	B	O
	64	256	8	64	4096

The hemagglutination inhibitory activity (HAI) of POL was unaffected by monosaccharides or disaccharides. However, melibiose and fetuin were able to inhibit POL activity, with minimum inhibitory concentrations of 100 and 6.25 mM, respectively. POL displayed its highest hemagglutination activity at pH 6 and 7, both registering at 100%. Conversely, its activity was negligible at pH 1, 2, 3, 10, 11, and 12, each recording 0% compared to the control. These findings indicate that POL activity remains stable up to 40 °C, decreasing to 50% at 60 °C, and ceasing entirely at 80 °C compared to the control. Furthermore, the impact of various metal cations on lectin activity was assessed. In Table S1, it is shown that the hemagglutination titer of the purified lectin was unaffected by the addition of Mg^2+^, K^2+^, Ca^2+^, Ba^2+^, Zn^2+^, Mn^2+^, and Al^3+^ ions. However, the activity of POL was dose-dependently inhibited upon the addition of Fe^3+^ (6–10 mM).

### Evaluation of the purified lectin cytotoxicity

The dose-response relationship for the cytotoxic effect of POL on both the Vero cell line and PBMCs is illustrated in Table S2. Additionally, the cytotoxicity of POL was evaluated on other cell lines, including Huh-7 and HepG2. It is noteworthy that the impact of POL on both normal and cancer cells followed a dose-dependent pattern. The data obtained from the experiments indicated that incubating POL with the Vero and PBMC cell lines showed minimal cytotoxic activity, as indicated in Table 3 by the 100% effective concentration (EC_100_) values of 3.14 and 2.96 µM, respectively. Furthermore, Table 3 presents the IC_50_ values of POL against the human hepatoma cancer cells HepG2 and Huh-7 used in this study, which were 68.20 and 38.21 µM, respectively. These findings emphasize the lack of significant cytotoxicity of POL towards normal cell lines under the tested conditions.

**Table 3:** The IC_50_ and EC_100_ values of POL against PBMCs, Vero, HepG2 and Huh-7 cell lines.

	Vero	PBMCs	HepG2	Huh-7
IC_50_ (µM) ± SEM**	80.35 ± 6.95^b^	123.70 ± 3.21^a^	68.20 ± 4.89^b^	38.21 ± 2.03^a^
EC_100_ (µM) ± SEM**	3.14 ± 1.76 ^b^	2.96 ± 1.22 ^a^	0.84 ± 0.11^a^	0.47 ± 0.06^b^

: EC_100_:  effective concentration at which 100% cell viability is maintained; IC_50_: concentration at which 50% inhibition of cancer cell growth is observed.

**The data are expressed as mean ± SEM (*n* = 3). Different letters indicate the significance at *p* < 0.05.

### Evaluation of antiviral activity of POL

#### The antiviral activity of the purified lectin against HCV and HBV

**Fig. 3:**
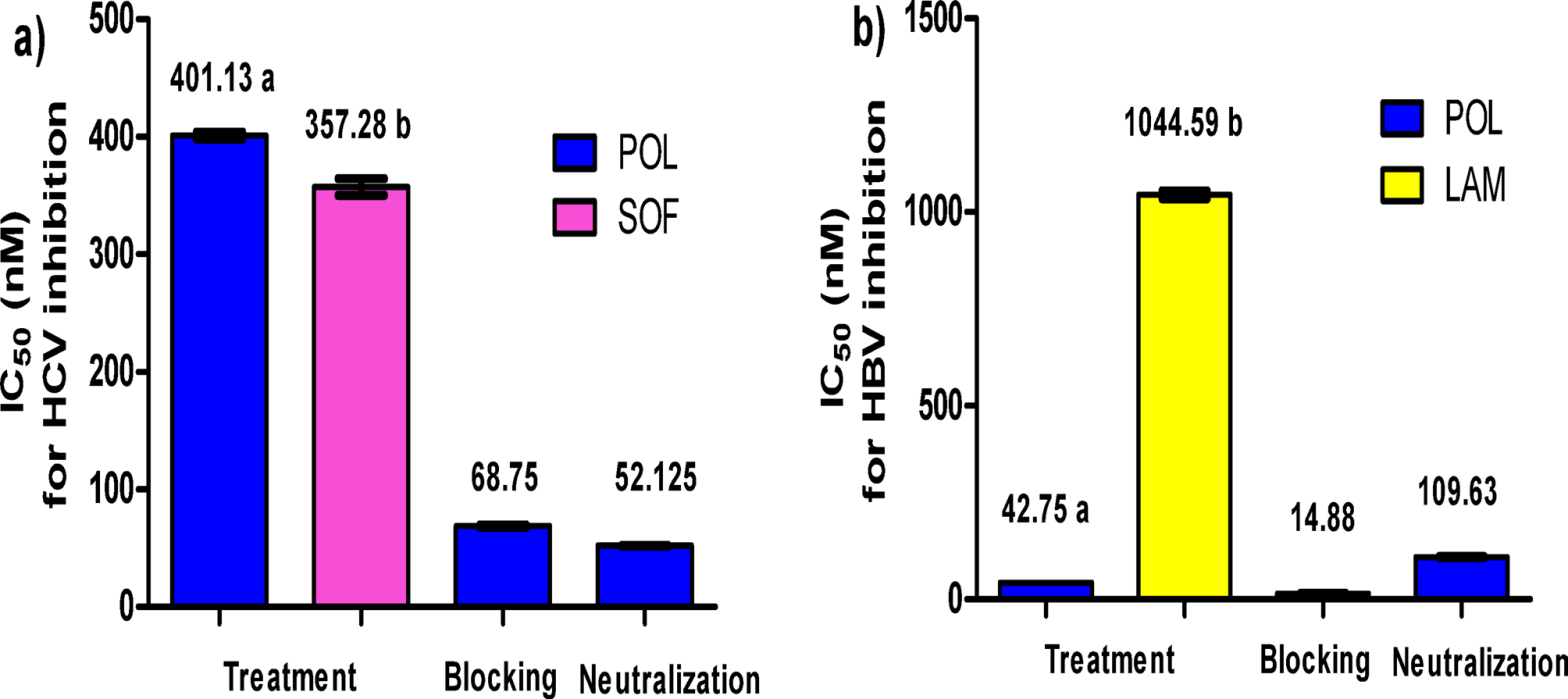
Inhibitory effect of lectin. (**a**) IC_50_ values of POL and SOF inhibitory effects on the HCV replication. (**b**) IC_50_ values of POL and LAM inhibitory effects on the HBV replication. Data are illustrated as mean ± SEM. Different letters indicate the significance at *p* < 0.05.

In Fig. 3a and Supplementary Table S3, it is demonstrated that POL exhibits promising anti-HCV effects, particularly when employed in blocking and neutralizing mechanisms. The inhibition percentages of the lectin showed dose-dependency. In the context of positive control infected Huh-7 cells, the HCV titer increased from 2.9 × 10^4^ to 3.3 × 10^6^ copies/mL. Notably, the application of 12.5 µM POL resulted in the complete prevention of HCV entry into Huh-7 cells. Impressively, POL displayed the most notable efficacy in the blocking and neutralizing mechanisms, as evidenced by its significantly lower IC_50_ concentrations of 68.75 and 52.125 nM, respectively, compared to the standard anti-HCV drug sofosbuvir (SOF). Unlike POL, sofosbuvir exhibited a high IC_50_ value of 357.28 nM in the treatment mechanism, without demonstrating any preventive effects in the blocking or neutralization mechanisms. Additionally, it is important to note that POL exhibited a notably high selectivity index (SI) value of 2360.69 in its neutralizing effect, indicating a substantial margin between protein cytotoxicity and antiviral efficacy. In Fig. 3b and Supplementary Table S4, POL demonstrated promising anti-HBV effects, displaying increased potency when engaging treatment and blocking mechanisms. Similarly, the percentages of lectin inhibition displayed a dependence on dosage. In the scenario involving positive control infected HepG2 cells, the HBV titer increased from 1.0 × 10^4^ to 6.69 × 10^5^ copies/ml. Intriguingly, the utilization of 12.5 µM POL exhibited the ability to prevent both HBV entry and replication within HepG2 cells. POL showcased its capability to prevent HBV replication and effectively hinder viral entry, as evidenced by its IC_50_ values of 42.75 and 14.88 nM, respectively. In comparison, the standard anti-HBV drug, lamivudine (LAM), possessed an IC_50_ of 1.04 µM, yet did not manifest any blocking or neutralization effects.

The effect of lectin on inhibiting the replication of HBV inside the HepG2 cell line was also studied in a qualitative way by using rapid diagnostic test (RDT) that detects both HBeAg and HBsAg. From Table 4 it shows the RDT results where 12.5 µM of POL showed negative results in both HBeAg and HBsAg. IC_50_, 50% inhibitory concentration against virus.

**Table 4:** Effect of POL on HBeAg and HBsAg production.

Concentration (µg/mL)	HBV treatment		HBV blocking		HBV neutralization	
	HBeAg	HBsAg	HBeAg	HBsAg	HBeAg	HBsAg
12.5	−	−	−	−	−	−
1.25	−	−	−	−	+	+
0.125	+	+	+	+	+	+

*HBeAg* hepatitis B e antigen, *HBsAg* hepatitis B surface antigen.

#### The mechanism of antiviral activity against HCV and HBV

**Fig. 4:**
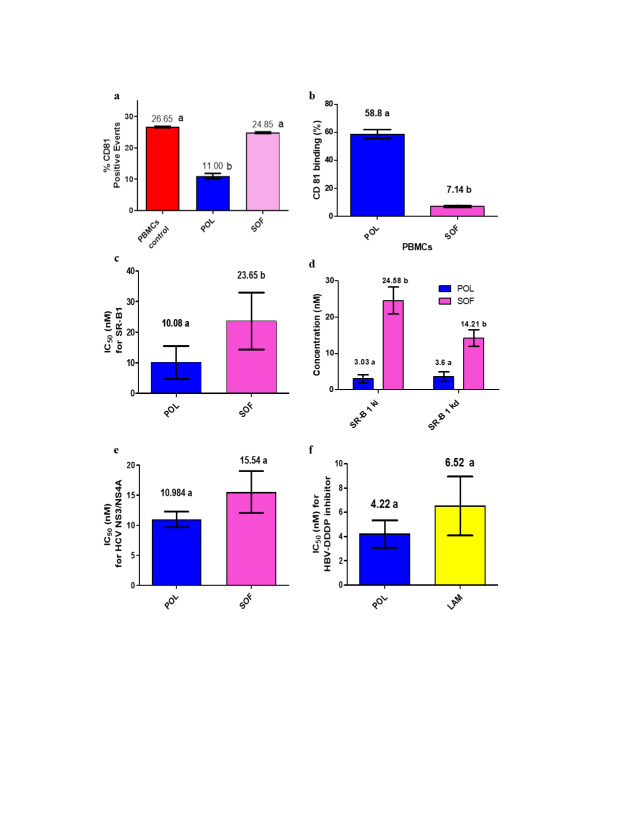
The antiviral mechanisms of POL against HBV and HCV. **(a)** Flow cytometry analysis of CD81 expression on the surface of normal PBMCs before and after the addition of lectin and SOF. **(b)** Flow cytometric analysis of the % CD81 in healthy PBMCs. **(c)** IC_50_ values of POL on the cellular scavenger receptor B type I (SR-B1) when compared with SOF. **(d)** Inhibition constant (Ki) and dissociation constant (Kd) values for binding of POL with SR-B1 receptor. **(e)** IC_50_ values of POL and SOF inhibitory effects on the HCV NS3/NS4A protease. **(f)** IC_50_ values of POL and LAM inhibitory effects on the HBV polymerase. Data are illustrated as mean ± SEM. Different letters indicate the significance at *p* < 0.05.

The expression of CD81 on the cell surface of PBMCs was demonstrated through flow cytometric analysis using the FITC-anti-CD81 antibody, as shown in Fig. 4a and Supplementary Table S5. After incubation with PBMCs, lectin led to varying degrees of reduction in the amounts of unbound CD81, indicating enhanced adherence to the receptor. Notably, POL exhibited superior binding capability compared to sofosbuvir, as illustrated in Fig. 4b. The calculated percentage of POL binding to CD81 on the PBMC cell surface was found to be 58.80% at a concentration of 12.5 µM. In direct contrast to SOF, POL exhibited a significantly heightened (*P* < 0.05) ability to bind with CD81. Figure 4c and Supplementary Table S6 provide a graphical representation of POL’s impact on the SR-B1 receptor of the HCV-host cell. This effect was observed to be dose-dependent, with experimentation involving various concentrations of the lectin. Interestingly, POL displayed a notably low IC_50_ value of 10.08 nM, which was statistically significant compared to SOF. To assess the affinity and efficacy of the studied lectin for SR-B1, both equilibrium competition and saturation binding methods were employed. The results, detailed in Fig. 4d and Supplementary Table S7, revealed that POL exhibited substantially low Ki and Kd values of 3.03 and 3.6 nM, respectively, underscoring a high affinity of binding with the receptor. The concentration-dependent suppression of HCV protease enzyme (NS3/NS4A) activity is highlighted in Fig. 4e and Supplementary Table S8. The findings revealed no significant disparity in the IC_50_ values between POL and SOF in terms of inhibitory efficiency, with recorded IC_50_ values of 10.98 and 15.54 nM, respectively. Moving to HBV polymerase, Fig. 4f and Supplementary Table S9 exhibit POL’s capacity to inhibit polymerase activity in a concentration-dependent manner. Despite variations in concentration, POL and LAM displayed non-statistically significant IC_50_ values of 4.22 and 6.52 nM, respectively.

### Protein modeling

**Fig. 5:**
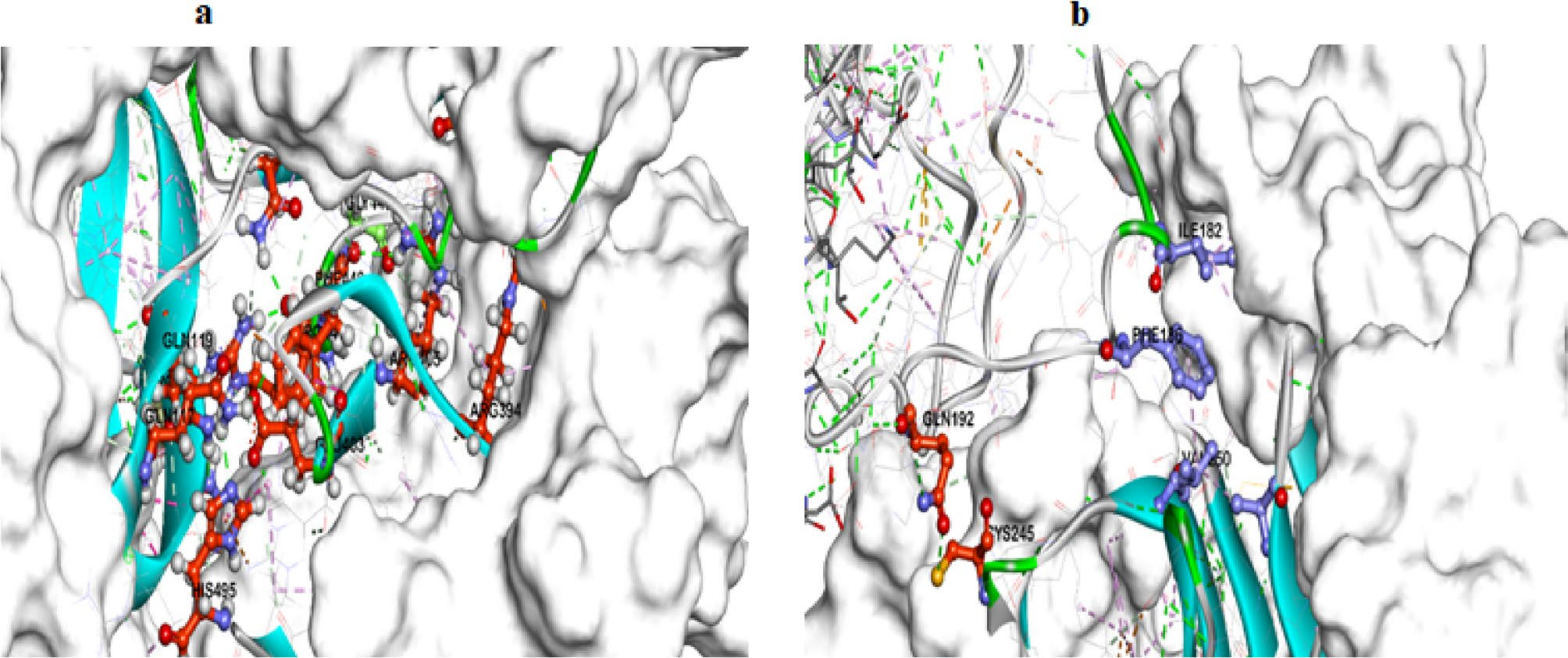
(**a**) SR-B1docked against POL; (**b**) Human CD81 docked against POL; hydrogen bonds are represented in green lines.

Protein-protein docking was studied to get a better understanding of the inhibitory functions of POL on early viral entry and replication. MOE 19.0901 software was used to perform the docking technique. The results of protein docking showed that the binding mode of POL (PDB code 6KBJ) with CD81, SR-B1, and HCV protease exhibited an energy binding of -130.55, -250.58, and − 108.45 kcal/mol^− 1^, respectively as shown in Table 5. The SR-B1 domain and POL formed four hydrogen bonds between Gln117, Gln119, Ser218, and Asn321 against His495, Glu403, Arg394 and Arg449, respectively as in Fig. 5a. On the other hand, CD81 as in Fig. 5b and HCV protease have a weak interaction with POL compared to its interaction with SR-B1.

**Table 5:** Molecular Docking of human CD81 (PDB code 1G8Q), SR-B1 (PDB code 7C00), and HCV protease (PDB code 1NS3).

Target receptor	PDB code of ligand*	Ligand	3D interaction	Docking Score	NO. Hydrogen bonds
	**CD81(1G8Q)**			**-130.55**	**1**
	**SR-B1 (7C00)**			**-250.58**	**4**
	**HCV protease (1NS3)**			**-108.45**	1

## Discussion

This study aimed to evaluate the antiviral effect of purified lectin from the edible mushroom, *Pleurotus ostreatus*. *P. ostreatus* mushrooms are among the most studied and cultivated edible mushrooms in the world. *P. ostreatus* lectin (POL) was partially purified and characterized from *P. ostreatus* mushrooms. In the present study, aqueous extracts were obtained from the fresh mushroom fruiting bodies, followed by ammonium sulfate precipitation using 30%, 60%, and 90% saturation. The protein content and hemagglutination titer were highest when 60% ammonium sulfate was used. Alcohol was also used as a precipitation method (data not shown), yielding approximately the same protein concentration as ammonium sulfate. However, alcohol masked the hemagglutination activity of the resultant protein. Detergents such as EDTA and Triton X-100 were not used because they alter hemagglutination detection and cause blood hemolysis^19^. Two chromatographic steps were involved in the lectin purification protocol: an ion exchange step using a Mono Q column and affinity chromatography using fetuin. Similarly, Lin and Tzi^20^ extracted antineoplastic melibiose-binding lectins from *Bauhinia variegata* seeds using an FPLC-Mono Q column. Other studies have purified lectins from the fruiting bodies of mushrooms using both anion and cation chromatographic columns, such as *Agrocybe cylindracea*^21^ and *Inocybe umbrinella*^22^. Zhang et al.^23^ noted that many isolated lectins can be adsorbed on both anion and cation chromatographic exchanger columns. These discrepancies may be attributed to variations in purification protocols, chromatography steps, or differences in lectin properties among mushroom species.

In this work, the purification of POL enabled a 32-fold purification of the lectin from the crude extract of mushroom fruiting bodies. This purification fold was higher than that reported in another study on *Ganoderma lucidum* mushrooms, which showed a 13.3-fold purification^24^. However, the result of the present work was lower than that reported by Brown et al.^25^ who purified lectins through three steps of chromatography and obtained a purification fold of 161 from *Inocybe umbrinella* mushroom. In this study, from 500 gm of mushroom fruiting body, about 1257 mg of POL, were purified. The recovery rate was 48%. Other studies showed yield of lectins equal to 35.71% from *Ganoderma lucidum*^24^25.3% from *Agrocybe cylindracea*^21^ and 20.8% from *Bauhinia variegate*^26^. In agreement with the current findings on POL, other studies have suggested that the native molecular mass of POL is 90 kDa, based on gel filtration results. This indicates that POL consists of two identical subunits of 45 kDa without S-S linkage^27,28^. The protein content of the crude extract from 2 g of oyster mushroom was 0.70 mg/mL. A study examining the protein content of crude extracts from 35 species of mushrooms using the Bradford method showed that the highest protein amount was observed in *Cantharellus subcibarius* at 1.567 mg/mL, and the lowest in *Cordyceps sinensis* at 0.131 mg/mL^29^. The highest hemagglutination activity (HA) was 4096 HU toward human blood group O, while the lowest was toward human blood group A. Conrad and Rüdiger found that POL specificity to human blood ABO groups was better than in rabbit erythrocytes^28^. Another study demonstrated that POL exhibited a slight preference for type O rather than type A and B^27^. These two studies are consistent with the results of this work. POL lost 50% of its hemagglutination activity at 60 °C, and the activity completely diminished at 80 °C, which is in agreement with the findings of Wang et al.^30^. The hemagglutination inhibition (HAI) assay was used to identify the carbohydrate specificity of the purified lectins. Different types of fungi can produce large quantities of saline-soluble, low-molecular-weight lectins that bind to glycoproteins like fetuin but not to monosaccharides. None of the tested simple sugars in this study inhibited the hemagglutination activity of the purified lectin at different concentrations ranging from 200 mM to 0.78 mM. This finding is consistent with Carrizo et al.^31^. POL hemagglutination activity was inhibited by 100 mM melibiose and 6.26 mM fetuin. Various studies have indicated that POL is selective for carbohydrates with a galactosyl moiety, such as N-acetyl-D-galactosamine. Melibiose, at a concentration of 40 mM, is the most powerful inhibitor of hemagglutination^28,30^. The inhibition of POL activity by melibiose in this study was consistent with the findings of Wang and Ng^32^although at a higher melibiose concentration. Lectins often show poor selectivity for monosaccharides, with association constants in the millimolar range, while their affinity for oligosaccharides is frequently significantly stronger, with association constants up to threefold higher. Surface plasmon resonance (SPR) experiments with various sugars revealed that Me-α-GalNAc and 2′-fucosyllactose exhibited the highest selectivity for lectins^33^. The three-dimensional structure of POL, as well as the characteristics of its interactions with carbohydrates, remain unclear despite being crystallized about 20 years ago^34^. The amount and composition of lectins vary depending on the harvest season, location, and age of the carpophore^35^.

There was no change in the hemagglutination (HA) of the lectin when incubated with divalent cations, while the activity of POL was slightly diminished with the Fe^3+^ trivalent cation. A study by Sun et al.^35^ showed that the hemagglutination titer of Boletus species hemagglutinin (BSH) was unchanged with K^+^, Cd^2+^, Cu^2+^, Mg^2+^, Mn^2+^, Zn^2+^, Al^3+^, and Fe^3+^ ions at concentrations of 1.25–10 mM but was diminished by Fe^2+^ at 5–10 mM. However, solutions of Mn^2+^, Zn^2+^, and Mg^2+^ at concentrations of 1–10 mM reduced the HA of Boletus edulis lectins to 50%. Moreover, a Ca^2+^ solution in the same concentration range did not affect the lectin’s activity^36^. Another study demonstrated that the hemagglutinating effect of POL was inhibited by Ca^2+^, Mg^2+^, Mn^2+^, and Fe^3+^ ions and potentiated by Al^3+^^30^. POL is considered a safe and non-cytotoxic lectin. The cell viability of the lectin was assessed on Vero and PBMC cells, showing no cytotoxic effects on the tested cell lines. Data from this study corroborated findings by Lugang et al.^37^ who demonstrated the safety and reversible non-cytotoxic antiproliferative effect of ABL. However, one study reported that the mushroom lectin with hemolytic and cytotoxic effects was a lectin from *Laetiporus sulfureus* (LSL), which has a β-trefoil scaffold model^38^. This lectin has a marked specificity toward lactose disaccharide, especially N-acetyllactosamine, and contains two sugar-binding sites^39^.

In this study, HCV- and HBV-positive sera were collected from individuals with naive HBV and HCV infections. Both patients’ sera and pooled sera from numerous HBV and HCV-infected donors were included^10^. The therapeutic objective for HCV and HBV is to block viral replication without causing side effects in normal cells; therefore, new safe natural agents are urgently required^40^. The viral inhibitory efficacy of the tested lectin was dose dependent. The results showed that POL exhibited various mechanisms for its antiviral effect. POL inhibits viral entry either by binding to host receptors or to viral spikes. Additionally, POL inhibits viral replication by inactivating viral replication enzymes. There was a significant difference between the IC_50_ values of the tested lectin and SOF in their ability to inhibit HCV infection, with a total viral load of 3.35 × 10^6^ ± 0.08 IU/L. Approximately 12.5 µM of POL can decrease the HCV viral load by about 92%. Furthermore, by binding to their receptors, 12.5 µM of POL was able to block nearly 100% of HCV from entering the host cells. GRFT, a mannose-binding lectin purified from red algae, has been demonstrated to have an anti-HCV effect in vitro and to mitigate HCV infection in vivo^41^ GRFT’s anti-HCV effect is mediated by preventing HCV from being transmitted directly from cell to cell. Additionally, GRFT binds to the sugars present on the viral spike proteins, thus affecting the binding between the viral spike protein and the host receptor CD81, preventing virus attachment and entry. The HCV cell culture system (with chimeric HCV H77/JFH1 virus) responded to the antiviral effect of GRFT with IC_50_ values of 87.2 and 223 ng/mL, respectively^41^. Carbohydrate-binding agents (CBAs) may be the first chemotherapeutics with a dual mechanism of antiviral action: first, through direct antiviral activity by binding to the glycans of the viral envelope and subsequently blocking virus entry; and second, through indirect (supplemental) antiviral action, resulting from the profound creation of deletions in the envelope glycan shield, thereby activating the immune system to act against deeply hidden immunogenic epitopes of the viral envelope^42^. Compounds that effectively block the earliest phases of viral propagation may qualify as prospective microbicidal agents with the potential to prevent the development of long-term infections^42^. Lectins might be utilized to prevent graft reinfection in HCV-infected liver transplant patients or to treat HCV-HIV coinfected individuals, where medication interactions with antiretrovirals are common. The wide antiviral spectrum, on the other hand, is a crucial aspect of CBA-based therapy strategies. They demonstrate that changes in the E1E2 envelope protein genes do not directly impart HCV resistance to CBAs but rather arise through an indirect route involving mutations in other viral proteins^43^.

Regarding HBV infection, POL was found to significantly limit the infection, resulting in a total viral load of 6.97 × 10^5^ ± 0.08 IU/L, with an IC_50_ value of 401.13 nM. Additionally, 12.5 µM of POL completely inhibited the release of HBsAg and HBeAg. Research by He et al.^44^ suggested a new mechanism for POL’s action on the immune response, proposing a therapeutic approach to break HBV tolerance. These findings indicate that POL influences HBV-specific humoral immune responses^44^. HBV entry inhibitory agents may help to neutralize residual viruses, protect naive hepatocytes from viral attack, and accelerate the natural clearance of infected cells^45^. The NTCP and HSPG, which attach to the HBV surface protein (preS1), are the only known HBV receptors. Combining nucleos(t)ide analogs (NAs) with entry inhibitors might expedite the clearance of contaminated cells. HBV entry blockers also prevent hepatitis D virus (HDV) infection^46^.

Research comparing the results of several cohort studies on the correlation between human mannan-binding lectin (MBL) and viral hepatitis concluded that low levels of serum MBL are linked to the development of HBV. Although the role of MBL in HCV disease remains unclear, they found an association between serum MBL levels and disease pathology, as well as the efficacy of HCV treatment^25^. A study by Bertaux et al.^47^ suggested that carbohydrate-binding agents (CBAs) inhibiting HIV entry could also extend their activity to other viral infections with highly glycosylated envelope glycoproteins, such as HCV. The current study defines the anti-HCV and anti-HBV activities of POL, generally inhibiting their cell entry and infection through neutralization and blocking. Additionally, the specific inhibitory effects of this lectin against CD81, SR-B1, HCV NS3/NS4A, and HBV polymerase were revealed. POL offers host resistance to virus entry and infection by blocking CD81 and SR-B1 receptors on host cells. CD81 is a 26-kDa member of the tetraspanin family, which binds to various membrane protein complexes and is responsible for multiple cellular functions^48^. CD81 was the first known HCV host receptor capable of binding to the viral envelope glycoprotein E2^49^. SR-B1 is an 82-kDa glycoprotein with two C and N-terminal domains and a large extracellular domain that separates them^50^. SR-B1 is primarily expressed in hepatocytes^50^. By interacting with the HCV virion’s associated lipoproteins and E2 glycoprotein, they help the virion’s attachment to the cellular surface^46^. The reduction in unbound CD81 levels was concentration-dependent on the lectin dose. A significantly higher (*P* < 0.05) binding efficiency to CD81 was detected with POL, followed by SOF. A study by Brown et al.^51^ demonstrated the ability of MBL to interact with the HCV envelope glycoproteins E1 and E2, resulting in virus neutralization and prevention of virus entry. Moreover, MBL activates the lectin-dependent complement pathway. The lectin cyanovirin-N (CV-N), a mannose-specific lectin from the cyanobacterium *Nostoc ellipsosporum*, also showed a potent antiviral effect with an IC_50_ of 1.6–17.6 nM against HCV infection. The anti-HCV activity of CV-N is mediated by its binding to glycoprotein E2, disrupting the interaction between E2 and CD81, thereby inhibiting HCV entry^52^. This study also found that POL’s binding to the SR-B1 receptor is dose-dependent. To detect the affinity of tested compounds to SR-B1, equilibrium competition (Ki) and saturation (Kd) binding assays were measured. Kd and Ki indicate the binding affinity between the receptor and the protein; smaller values indicate better affinity. According to the results, POL had significantly low Ki and Kd values, indicating a high binding affinity with SR-B1.

With respect to the HCV NS3/NS4A protease, the data showed a non-significant but closely related inhibitory efficiency of POL and SOF. The NS3/NS4A enzyme is crucial for HCV replication because it regulates viral genomic expression. To unwind and bind the viral RNA genome, NS3 possesses helicase/ATPase activity in its C-terminal portion, as well as NTPase and serine protease activity in its N-terminal portion. The enzyme complex NS3/NS4A is integral to HCV pathogenesis, requiring NS4A as a co-factor. Because this enzyme lacks a proofreading role, numerous mutations arise during transcription, leading to drug resistance. Due to the critical role of NS3/NS4A in HCV replication, it is a potential target for anti-HCV medications^53^. POL and LAM exhibit significant inhibitory effects on HBV polymerase. HBV replication primarily relies on HBV polymerase, the sole viral protein with enzymatic function. This polymerase enzyme comprises three functional domains: RH, T, and TP (from C-terminus to N-terminus). It participates in various stages of the viral replication cycle, including encapsidation (or packaging), RNA binding, template switching, RNA protein priming, RNA degradation, and DNA synthesis. Consequently, promising anti-HBV drugs target this enzyme^54^. The current study reported that the lectin examined demonstrated significant dual antiviral efficacy against HCV and HBV by inhibiting HCV NS3/NS4A protease and HBV polymerase, resulting in the suppression of HCV and HBV replication. POL demonstrated remarkable potency against both HCV and HBV. The tested lectin inhibited viral entry in the early stages of infection as well as essential replication enzymes. Furthermore, these lectins are safe and non-toxic to normal cell lines, suggesting that they may serve as a foundation for the development of safe and efficient antiviral medications.

The structural characteristics of lectins are responsible for the carbohydrate-binding specificity of lectins and thus their uniqueness^55^. The crystal structure of POL contains a single polypeptide chain forming two tandem repeats of β-jelly roll domains (domain A and domain B)^55^. Computational results of POL with CD81, SR-B1, and HCV protease revealed different types of interactions. Docking data demonstrated that POL formed more stable hydrogen bonds with SR-B1 than with CD81 and HCV protease. The data obtained from in-silico models show only changes of smaller magnitudes compared to those measured in vitro.

## Conclusion

The purified lectin from *Pleurotus ostreatus* mushroom (POL) demonstrates an in vitro safety profile alongside potent antiviral effects against the studied viruses. An investigation into its capacity to impede early viral entry has revealed that POL exhibits a high binding affinity to CD81 and SR-B1 receptors. Additionally, POL possesses the capability to curtail viral replication by inhibiting key viral enzymes, including HCV NS3/NS4A, HBV polymerase, and others. Furthermore, docking data support the notion that POL may engage in interactions with CD81 and SR-B1 receptors. These findings underscore the promising antiviral potential of the studied lectin against a spectrum of viruses, including HBV and HCV, suggesting its candidacy as a prospective therapeutic agent against these pathogens. While these results are encouraging, further research is imperative to elucidate the precise mechanisms underpinning POL’s antiviral effects and to assess its safety and efficacy in clinical contexts. Moreover, exploring potential applications of lectins in preventing viral reinfections or as adjunct therapy for co-infected patients holds considerable promise. Additionally, continued investigation into the variations in lectin composition and their implications for antiviral activity remains a valuable avenue of research. While this study includes direct viral infection assays and investigates specific antiviral mechanisms of POL through ELISA and in silico analyses, it is limited by the absence of molecular-level investigations—such as gene expression profiling—that would further clarify the underlying mechanisms. Additionally, in vivo validation could not be performed due to the unavailability of suitable HCV and HBV animal models in our laboratory. Nonetheless, we recognize the importance of these approaches and aim to pursue them in future studies using advanced models such as transgenic or chimeric mice.

## Materials and methods

### Extraction and purification of lectin from *P. ostreatus* mushroom

About 500 g of fresh *P. ostreatus* mushrooms were sourced from the College of Agriculture, Saba Basha, Alexandria University, Egypt. The species was identified by Prof. Ahmed M. Abdel-Azeem, Botany and Microbiology Department, Faculty of Science, Suez Canal University, through morphological and microscopic examination. The identified specimen was deposited in the Fungarium of Suez Canal University (https://ccinfo.wdcm.org/details?regnum=1180) under the accession number SCUF000000812 for *Pleurotus ostreatus*. After that, fresh *P. ostreatus* mushrooms were washed with distilled water, then cut into small pieces, and homogenized in a blender for 2 min at 5000 rounds per minute (rpm) with 500 milliliters of cold phosphate-buffered saline (PBS) at pH 7.4, containing 2 millimolar protease inhibiting cocktail, with all processes conducted at 4 °C. The homogenate was left to stand overnight under constant stirring. Subsequently, centrifugation was performed at 8000 rpm for 30 min. The supernatant was then collected and filtered using cellulose filter paper to remove any insoluble materials^56^. Salting out was achieved through ammonium sulfate ((NH_4_)_2_SO_4_) fractionation precipitation. Three fractions of solid (NH_4_)_2_SO_4_ were added to the supernatant: 30%, 60%, and 90%. Initially, the supernatant was gradually saturated with solid (NH_4_)_2_SO_4_ until it reached 30%, with all (NH_4_)_2_SO_4_ dissolved. The mixture was then gently stirred overnight to prevent protein denaturation. The following day, the mixture underwent centrifugation at 12,000 rpm for 30 min. After centrifugation, the insoluble pellet was gathered and dissolved in 0.02 M PBS (pH 7.4) containing 2 mM PMSF. Subsequently, additional precipitation of the supernatant occurred by adding (NH_4_)_2_SO_4_ powder to attain a final concentration of 60%. The same procedure was repeated to achieve a concentration of 90% ammonium sulfate, following the previously described method^57^. The resulting precipitate was then dialyzed. The dialyzed solution underwent filtration using a syringe filter with 0.45 μm pores before being loaded onto chromatographic columns.

The diethylaminoethyl-cellulose (DEAE-C) column (1.6 × 25 cm) was washed and pre-equilibrated with 0.02 M PB (pH 7.4). The clear supernatant was applied to the column to adsorb all the yellow-colored material. The colorless effluent with hemagglutination activity was collected. Fast protein liquid chromatography (FPLC) was employed to separate the target protein from the mixture. The effluent was loaded onto a Mono Q 5/50GL column chromatography, a strong anion exchanger for high-performance ion exchange chromatography, on AKTA prime plus FPLC. The column was washed and pre-equilibrated with 0.05 M PB (pH 7.4), and then, using a gradient of NaCl from 0.0 to 1.0 M in the same buffer at a flow rate of 1.0 mL/min, the bound proteins were eluted. Fractions were collected in (4 mL/tube). Protein-containing fractions were combined and immediately dialyzed with PBS buffer. A combination of ion-exchange columns with affinity columns was utilized for the further purification of target protein molecules.

The Fetuin-Sepharose CL-6B affinity gel was prepared as follows: Cyanogen bromide-activated Sepharose CL-6B was swollen by adding distilled water. Then, 50 g of the swollen gel were weighed and mixed with 50 milliliters of bovine fetuin solution. This solution was prepared by adding 500 milligrams of fetuin to a solution containing 0.5 M sodium chloride, 0.1 M sodium bicarbonate, and adjusting the final coupling pH to 8.3. The coupling reaction was covered and allowed to proceed for 4 h at room temperature with gentle shaking. Subsequently, 0.1 M aminoethanol was used to block the excess reactive sites, and the reaction was left to proceed overnight at 4 °C.

The gel was washed successively with 400 milliliters of the coupling buffer, followed by 400 milliliters of 3 M sodium thiocyanate at pH 7.5. Afterward, the gel was equilibrated with 50 mM PBS at pH 7.4 and stored at 4 °C to prevent contamination, fetuin-Sepharose CL6B was heated at 65 °C for 16 h. The lectin-eluted fractions were further purified using the fetuin column. The column was washed with 50 mM PBS at pH 7.4, and the fraction was loaded into the column. Subsequently, the adsorbed proteins were desorbed using a gradient of NaCl (0.25, 0.5, 0.75, and 1 M NaCl) in 50 mM glycine-HCl buffer at pH 3 with a flow rate of 1.0 mL/min, with a fraction size of 5.0 mL, divided into 4 fractions (fractions 1 to 4). The eluted fraction was neutralized using a neutralizing solution (1 M Tris HCl pH 8, 1.5 M NaCl, and 1 mM EDTA). All eluted fractions were dialyzed to remove excess salt, and the lectin solution was concentrated by lyophilization.

Subsequently, different fractions were assayed for the presence of lectin using a hemagglutination assay. The protein concentration was assessed using the Bradford technique. However, carbohydrate contents were determined using the phenol sulfuric acid procedure.

### Characterization of the purified lectin

In the hemagglutination assay, blood grouping agglutination tests antibodies were used to determine the activity of the purified lectin. Blood was centrifuged at 1500 rpm at 25 °C, then erythrocyte preparations were made fresh by washing the RBCs three times with 5 volumes of 50 mM PB (pH 7.4) and diluting to 2% suspensions (v/v) in the same buffer. The blood was then stored in preserving medium at 4 °C for up to 2 weeks. The hemagglutination method was conducted in 96-well microtiter U-shaped plates with a final volume of 50 µl. A serial two-fold dilution of a 25 µL lectin sample with an equal volume of 50 mM PB (pH 7.4) was prepared. Then, 25 µL of RBC suspension was added to each well and incubated for 30 min at 37 °C and another 30 min at room temperature. The reciprocal of the highest sample dilution that totally agglutinates RBCs was used to calculate the hemagglutination titer. Each assay was repeated three times and evaluated with ConA as a standard^58^.

The SDS-PAGE technique was utilized to determine the molecular mass of proteins and to assess their homogeneity and purity levels. Specifically, SDS-PAGE was conducted using 15% polyacrylamide gels. To assess the inhibitory activity of sugars and glycoproteins on lectin-mediated hemagglutination, a range of compounds including D-glucose, D-mannose, D-fructose, lactose, D-melibiose, dextrin, amylose, D-galactose, sorbitol, sucrose, D-maltose, N-acetyl glucosamine, mannitol, cellobiose, fetuin, inulin, pectin, and L-rhamnose were tested. A serial two-fold dilution of 25 µL of 200 mM of various carbohydrates or glycoproteins was mixed with an equal volume of 50 mM phosphate buffer in 96-well microtiter U-shaped plates (pH 7.4). Subsequently, 25 µL of each purified lectin (64 HU) was added to all wells and incubated at 37ºC for 60 min. After this incubation period, 50 µL of erythrocytes were added and incubated for an additional 60 min. The inhibitory efficacy was determined as the lowest carbohydrate or glycoprotein concentration that completely inhibited the hemagglutination titer of the purified lectin.

To investigate the effect of pH on hemagglutination activity, the hemagglutination activity of the purified lectin was measured after dialysis against buffers with various pH values. The buffers used included HCl-KCl buffer (pH 1.0 and 2.0), sodium citrate buffer (pH 3.0, 4.0, and 5.0), sodium phosphate buffer (pH 6.0 and 7.0), Tris-HCl buffer (pH 8.0), sodium bicarbonate buffer (pH 9.0 and 10), and glycine-NaOH buffer (pH 11.0 and 12.0). Each 500 µL aliquot of the purified lectin in 50 mM phosphate buffer (pH 7.4) was dialyzed against 500 mL of 50 mM buffer with various pH values from 1 to 12 overnight. The dialyzable fractions were then neutralized with 50 µL of a neutralizing buffer.

The lectin’s optimum temperature was determined by measuring its hemagglutination activity at different temperatures (20, 40, 60, 80, and 100ºC). Each 500 µL aliquot of the purified lectin in 50 mM phosphate buffer (pH 7.4) was incubated at different temperatures for various time intervals (10, 20, and 30 min). After cooling the samples to room temperature, they were centrifuged at 5,000 rpm for 5 min to remove precipitate. To investigate the effect of divalent cations on the lectin’s hemagglutination activity, 500 µL aliquots of the purified lectin were dialyzed with 20 mM phosphate buffer (pH 7.4) containing different concentrations (2–10 mM) of various cations (KI, KCl, CaCl_2_, ZnCl_2_, MgCl_2_, BaCl_2_, MnCl_2_, AlCl_3_, and FeCl_3_) in separate bottles at 4 °C overnight. The mixture was then incubated, and hemagglutination activity was assessed as described previously. Control samples were incubated with an equal volume of 20 mM phosphate buffer (pH 7.4) at 4 °C. Lectin activity was expressed as a percentage of relative activity compared to the control.

### Cell lines and media

Peripheral blood mononuclear cells (PBMCs) and Vero cell lines were utilized in this study to assess the cytotoxicity of the purified lectin. Vero, Huh-7, and HepG2 cell lines were sourced from the American Type Culture Collection (ATCC, USA). Specifically, Huh-7 and HepG2 cell lines were employed for studying hepatitis C virus (HCV) and hepatitis B virus (HBV), respectively. PBMCs were isolated by collecting 4 mL of peripheral blood samples from a single healthy human volunteer in heparinized vials. All human and animal blood samples were collected strictly in accordance with the relevant animal care policies, as mandated by international standards. All healthy donors of blood gave us their written informed consent prior to participation. The study was approved by the Research Ethical Committee of the City of Scientific Research and Technological Applications (SRTA-City), and all relevant laws and regulations were followed during the proceedings. Subsequently, in a 10 mL centrifuge tube, Ficoll Histopaque media (4 mL) was added. The blood was gently layered onto the Ficoll Histopaque media solution and immediately centrifuged for 30 min at 1200 rpm, zero gradients at 25 °C in a swing-out bucket rotor. The resulting whitish buffy coat (approximately 1.0 mL comprising PBMCs) formed at the interface was promptly aspirated. This aspirated layer was then washed with 10 mL of supplemented media before being centrifuged twice at 2000 rpm and 25 °C for 10 min. The resulting pellet of cells was resuspended in 1.0 mL of complete media. Cell counts were performed, and the cells were subsequently diluted to the desired concentration using complete media.

### Assay of the cytotoxic effect of the purified lectin using MTT method

The cytotoxic activity of POL was assessed against both normal (Vero and PBMCs) and cancer (HepG2 and Huh-7) cell lines using the MTT assay. Cells were initially seeded at a density of 3 × 104 cells/mL in 200 µL of appropriate media per well in a 96-well cell culture plate. The plates were then placed in a 37ºC incubator with a humidified atmosphere containing 5% CO_2_ and 95% air. Following incubation, cells were exposed to various concentrations (25, 18.75, 12.5, 6.25, 3.125 µM) of POL in triplicate and further incubated for 72 h at 37 °C in 5% CO_2_ and 95% air. Daily observations were made to assess cell viability and adherence. Subsequently, 100 µL of 0.5 mg/mL MTT solution was added to each well and incubated at 37 °C for 5 h. Formed formazan crystals were dissolved by adding 200 µL of DMSO to each well. Optical density was then measured at two different wavelengths, 570 and 630 nm. The percentage of viability compared to untreated cells was calculated as follows:

1 $${\text{Cell}}\;{\text{viability}}\;\left( \% \right)={\text{Mean}}\;{\text{OD}}/{\text{Control}}\;\left( {{\text{untreated}}\;{\text{cells}}} \right){\text{ }}OD \times 100.$$.

Calculations were performed and the safe concentration (EC_100_) and EC_50_ was determined using Graph Pad prism 5.

### Evaluation of antiviral activity of POL

#### Establishment of viral host cells and viral infected serum samples

Huh-7 and HepG2 were employed in this study as HCV and HBV target cells, respectively. The effect of lectin on both Huh-7 and HepG2 human cancer cell lines was assayed using MTT as mentioned before in the cytotoxicity assay. The IC_50_ was calculated using Graph pad prism 5.

#### Investigation of the anti-HCV and anti-HBV modes of action of POL

The anti-replicative (treatment), direct virucidal (neutralization), and anti-adsorptive (blocking) effects of lectin on HCV- and HBV-infected hepatocyte cells were investigated. Huh-7 (1 × 10^6^ cells) and HepG2 (1 × 10^6^ cells) were seeded in 6-well plates in DMEM and RPMI medium, respectively. They were incubated in a CO_2_ incubator for 24 h at 37ºC, 5% CO_2_ and 95% humidity. The next day, after cells had reached 70–80% confluence, the old media was discarded, the cells washed, and the three mechanisms (blocking, neutralization, and treatment) were applied. An additional step for the HepG2 cell line before its infection with HBV was that the cells were treated with 4% PEG 6000 and 1.5% DMSO and left overnight before infection.

#### The effect of POL on the intracellular replication of HCV or HBV infection (treatment)

The seeded cells were treated with 1.0 mL serum infected with HCV or HBV for two h at 37ºC, 5% CO_2_ and 95% humidity. Then, cells were washed and 1.0 mL of three concentrations of the purified lectin (12.5, 1.25. and 0.125 µM) were added. Cells were incubated at 37ºC for 72 h with 5% CO_2_ and 95% humidity. After 72 h, the supernatant was collected, and the cells were detached for further analysis^59^.

#### Neutralizing effect of POL against HCV and HBV

One mL of infected HCV or HBV serum with the same concentrations was added to 1.0 mL of three concentrations of the purified lectin (12.5, 1.25. and 0.125 µM) and kept at room temperature for two h. This mixture of infected serum and lectin was added to the cells for two h. Cells were washed then, incubated for 72 h at 37ºC with 5% CO_2_ and 95% humidity. Steps were completed as mentioned in the previous step.

#### Blocking activity of POL against HCV and HBV

Each well received 1.0 mL of three concentrations of POL (12.5, 1.25, and 0.125 µM), which were then incubated for 2.0 h. Cells were washed, and then 1.0 mL of infected HCV or HBV serum was added for 2.0 h. Subsequently, cells were washed again and incubated for 72 h at 37ºC, 5% CO_2_, and 95% humidity. Two control wells were included and incubated under similar conditions: positive (infected cells without treatment) and negative (uninfected cells) (37,38). Sofosbuvir (SOF) served as the standard treatment for HCV, while lamivudine (LAM) was used for HBV. After 72 h of incubation, supernatant was collected for further analysis, and cells were washed and harvested. To evaluate the antiviral activity of POL against HCV and HBV, real-time PCR (Q-PCR) was employed. Q-PCR enabled the assessment of lectin’s antiviral activity by measuring the virus titer in infected cells. HCV-RNA and HBV-DNA nucleic acid extractions were carried out using the QIAamp DNA Blood Mini Kit and the RNeasy Mini Kit, respectively. For viral genome amplification and detection, ready-to-use q-PCR kits for HCV and HBV were utilized, following the kit protocol. The relative activity (%) of lectin was calculated using the following formula:

2 $${\text{Relative}}\;{\text{activity }}\% =\left( {{\text{A}} - {\text{B}}} \right)/{\text{A }} \times 100$$.

Where, A is the count of positive control, B is the count of tested proteins. The lectin’s capacity to suppress both HBsAg and HBeAg was investigated in the supernatant of HBV-infected cells using a rapid diagnostic test (RDT).

#### The effect of the purified lectin on the early viral entry

The ability of POL to block CD81 was assayed with flow cytometry using an anti-CD81-FITC antibody. Briefly 90 µl (1 × 10^6^ cells) of PBMCs were incubated for 2 h with 12.5 µM of POL. The cell suspension was then inoculated with 10 µL of the antibody solution and incubated at 4 °C for 30 min. The mixture was then completed to 1.0 mL with PBS, centrifuged at 2000 rpm for 5 min, and the cells dispersed in 2 mL of PBS before being identified by the Fluorescein isothiocyanate (FITC) signal detector (FL1). The ability of POL to block SR-B1 was examined using the Tag-lite™ receptor-ligand binding assay following the inserted protocol. Tag-lite™ is a cell based homogeneous time-resolved fluorescence (HTRF) customized assay kit that includes plasmids engineered to express tagged cell lines, fluorescent donor and acceptor reagents, labeling mediums, and some components that can be considered as standards. This technique combines the HTRF and self-labeling protein tag (SNAP-tag) technologies to create a cellular screening platform. The fluorescence-labeled SR-B1 ligand was combined with the terbium (Tb) cryptate derivative (SNAP-Lumi4-Tb)-labeled SR-B1-expressed cells. The fluorescence was monitored at zero and 30 min using an HTRF reader after varied amounts (125, 12.5, 1.25, and 0.125 nM) of both POL and SOF were applied. Saturation and competitive binding tests can both benefit from tag-lite technology. The saturation binding technology assesses the fluorescent ligand’s non-specific and total binding at equilibrium, and then the specific binding of the examined proteins was measured as the difference. The amount of protein that occupied 50% of the total receptor binding sites, dissociation constant (Kd), was calculated. The examined substance was titrated into a solution containing a given number of cells and a fixed amount of fluorescent ligand to quantify the inhibition constant (Ki) in the competitive binding experiment. The Kd and Ki values correspond to the receptor’s affinity for the labeled or unlabeled ligand, respectively.

#### Effect of the purified lectin on HCV-non-structural 3/ non-structural 4 A (NS3/NS4A) protease activity

HCV NS3 is a serine protease that looks like chymotrypsin. It needs a cofactor, a 54-amino-acid NS4 protein, to function properly. HCV NS3/4A protease is an essential enzyme for viral NS polyprotein cleavage at the NS3-NS4A, NS4A-NS4B, NS4B-NS5A, and NS5A-NS5B sites. Because the NS3/4A protease is required for viral replication and the production of infectious viral particles, it has been identified as a promising target for anti-HCV treatment^60^. The inhibiting activity of POL and SOF at various amounts (125, 12.5, 1.25, and 0.125 nM) on HCV-NS3/NS4A protease was assayed by Senso Lyte^®^ 490 HCV-Protease Assay Kit, and recombinant HCV NS3/NS4A protease genotype 1b from AnaSpec^®^, as described in the inserted manual. A fluorometric method is based on fluorescence resonance energy transfer (FRET), which involves incubating a labeled-fluorescence peptide substrate containing a sequence derived from the NS4A/NS4B cleavage site with the NS3/NS4A protease for 30 min at 37 °C. The fluorescence was produced upon proteolysis, and its strength was quantified using a fluorescent microplate reader at an excitation/emission rate of λex = 340 nm and λem = 490 nm. The control reaction (highest enzymatic activity) was performed in the experiments that use the enzyme without any of the components being examined. For the IC_50_ values (the amount of the tested proteins that produced 50% inhibitory effect) to be calculated from the dose-response curve, the inhibition rate of the enzyme was reported as a percent of inhibition^60^.

#### Effect of POL on the HBV polymerase

The modified Hirschman et al.^61^ method was used to determine the inhibitory power of POL (125, 12.5, 1.25, and 0.125 nM) on HBV polymerase activity when compared to LAM. A combination of 21 µl of the studied virus suspension, 7 µl of each concentration of investigated lectin or LAM, 340 mM KCl, 34 mM MgCl_2_, and 0.4% Nonidet P-40 (nonionic detergent) were used in the enzymatic process. Furthermore, 42 mM Tris-HCl (pH 7.5), 22 mM β-mercaptoethanol, and 70 mM of each of the 1µCi radiolabeled [3 H-TTP] deoxynucleotides were supplied. The newly synthesized DNA was then moved to filter paper discs and precipitated with 5% trichloroacetic acid before being quantified using a scintillation counter. Using GraphPad software, the inhibitory rate for HBV polymerase activity was utilized to calculate the IC_50_ values (the concentration of POL or LAM that inhibits polymerases by 50%).

### Method of Docking process

After choosing the protein target site, some processes should have been done to give insights into the molecular binding modes of the ligands inside the pockets of POL with PDB codes: 6KBJ, respectively, (https://www.rcsb.org) by using molecular operating environment (MOE) 19.0901 Software. Water molecules were initially eliminated from the compounds. After that, using the protein report and utility, as well as the clean protein options, crystallographic abnormalities and unfilled valence atoms were repaired. MMFF94 force fields were used to reduce protein energy. A fixed atom constraint was used to create the stiff structure of the protein. Then, the minimized structures were prepared for docking using a prepared protein protocol. The CDOCKER protocol was used to carry out the molecular docking process. During the refinement, the receptor (studied lectin) was kept rigid while the ligands were permitted to be flexible. Every molecule was given ten alternative interaction postures with the protein. The best-fitted postures with the active site are then docked scores (CDOCKER interaction energy) and the 3D view was generated by Discovery Studio 2019 Client software. All these methods were employed to estimate the suggested binding mode, affinity, preferred orientation of each docking posture, and binding free energy (ΔG).

### Statistical analysis

SPSS version 16 was used to analyze the data, which was represented as mean SEM. Duncan’s test was used to compare the mean values using one-way analysis of variance (ANOVA), and the significant value was set at (*p* < 0.05). The GraphPad Prism program version 5 was used to calculate the IC_50_, EC_50_, and EC_100_ values.

## Electronic supplementary material

Below is the link to the electronic supplementary material.

Supplementary Material 1

## Data Availability

All data generated or analysed during this study are included in this published article and supplementary information files.
