# Supplementary material for: Biochemical characterization of a novel purified lectin extracted from Pleurotus ostreatus mushroom for its antiviral activity
Source: Sci Rep. 2025 Jul 31;15:27893. doi: 10.1038/s41598-025-09967-z (PMC12311003; doi:10.1038/s41598-025-09967-z)
Supplement: Supplementary file 1 — Supplementary Material 1 [file 41598_2025_9967_MOESM1_ESM.docx]

**Supplementary file of:** **Antiviral Effect of a Novel Lectin Extracted from *Pleurotus ostreatus* Mushroom on Hepatitis C Virus and Hepatitis B Virus.**

**Table S1.** Effects of different cations (from 2 mM to 10 mM) on the HAI of lectin (POL)*

| **Cations** | **Hemagglutination activity of POL** |
| --- | --- |
| **MgCl_2_** | + |
| **KCl** | + |
| **CaCl_2_** | + |
| **BaCl_2_** | + |
| **ZnCl_2_** | + |
| **MnCl_2_** | + |
| **KI** | + |
| **AlCl_3_** | + |
| **FeCl_3_** | - (at conc. higher than 6 mM) |

* Initial hemagglutinating titer was 64 hemagglutinating units.

−, inhibition of hemagglutinating titer of the purified lectin; +, no inhibition of hemagglutinating titer of the purified lectin.

**Table S2.** Cell viability percentage and EC_50_/IC_50_ values of POL against Vero, PBMCs, HepG2 and Huh-7 cell lines*

| **Concentration (µM)** | **Cell viability (%) of POL** | | | |
| --- | --- | --- | --- | --- |
|  | **Vero** | **PBMCs** | **HepG2** | **Huh-7** |
| **25** | 75.25 | 86.81 | 70.39 | 50.48 |
| **18.75** | 79.88 | 92.74 | 81.28 | 75.95 |
| **12.5** | 90.85 | 93.01 | 89.94 | 86.82 |
| **6.25** | 98.24 | 100.79 | 93.40 | 90.54 |
| **3.125** | 100 | 113.28 | 97.87 | 95.48 |

* POL: *P. ostreatus* lectin.

**Table S3.** Inhibition percentage of HCV by the purified lectins on Huh-7 cell line*

| **Concentration (µM)** | **HCV treatment**  **(% inhibition)** | | **HCV blocking**  **(%inhibition)** | | **HCV neutralization**  **(% inhibition)** | |
| --- | --- | --- | --- | --- | --- | --- |
|  | **POL** | **SOF** | **POL** | **SOF** | **POL** | **SOF** |
| **12.5** | 92.07 | 73.79 | 99.97 | 0.044 | 96.41 | 0.019 |
| **1.25** | 88.17 | 35.66 | 58.88 | 0 | 80.84 | 0 |
| **0.125** | 8.42 | 12.04 | 52.85 | 0 | 60.63 | 0 |
| **IC_50_ (nM)** **± SEM **** | 401.13  ± 2.89 ^b^ | 357.28  ± 6.93 ^a^ | 68.75  ± 0.87 | Non | 52.125  ± 0.32 | Non |
| **SI***** | 308.48 |  | 1800.58 |  | 2360.69 |  |

* POL: *P. ostreatus* lectin and SOF: Sofosbuvir.

**Data are expressed as mean ± SEM. Different letters are statistically distinctive at p < 0.05.

***SI= selectivity index (EC_50_/IC_50_).

**Table S4.** Inhibition percentage of HBV by the purified lectins on HepG2 cell line*

| **Concentration (µM)** | **HBV treatment**  **(% inhibition)** | | **HBV blocking**  **(% inhibition)** | | **HBV neutralization**  **(% inhibition)** | |
| --- | --- | --- | --- | --- | --- | --- |
|  | **POL** | **LAM** | **POL** | **LAM** | **POL** | **LAM** |
| **12.5** | 100 | 75.92 | 99.26 | 0.019 | 97.67 | 0.015 |
| **1.25** | 75.67 | 55.53 | 88.56 | 0.014 | 89.49 | 0 |
| **0.125** | 63.51 | 21.28 | 76.01 | 0 | 50.19 | 0 |
| **IC_50_ (nM) ± SEM **** | 42.75± 0.16^a^ | 1044.59± 9.83^b^ | 14.88± 0.2 | None | 109.63± 1.13 | None |
| **SI***** | 2896.96 |  | 8313.17 |  | 1134.86 |  |

* POL: *P. ostreatus* lectin and LAM: Lamivudin

**Data are expressed as mean ± SEM. Different letters are statistically distinctive at at p < 0.05.

***SI= selectivity index (EC_50_/IC_50_).

Table S5. The percentage of the purified lectins and SOF binding to CD81 on PBMC*

|  | **PBMCs** | **POL** | **SOF** |
| --- | --- | --- | --- |
| **%Gating** | 26.75 | 11.00 | 24.79 |
| **Mean %CD81 binding ± SEM**** |  | 58.80 ± 3.18 ^a^ | 7.14 ± 0.77 ^b^ |

* POL: *P. ostreatus* lectin, PBMC: peripheral blood mononuclear cells SOF: sofosbuvir.

**The data are expressed as mean ± SEM (n=3). Different letters indicate the significance at p < 0.05.

Table S6. Blocking effect and IC_50_ values of POL on the cellular scavenger receptor B type I (SR-B1) when compared with SOF*

| **Concentration (nM)** | **SR-B1 blocking (%)** | |
| --- | --- | --- |
|  | **POL** | **SOF** |
| **125** | 77.53 | 76.71 |
| **12.5** | 54.07 | 60.60 |
| **1.25** | 23.90 | 32.70 |
| **0.125** | 3.42 | 1.26 |
| **IC_50_ (nM) ± SEM**** | 10.08 ± 5.4^a^ | 23.65 ± 9.36^b^ |

* POL: *P. ostreatus* lectin and SOF: Sofosbuvir.

**The data are expressed as mean ± SEM (n=3) and different letters indicate the significance at p < 0.05.

**Table S7.** Inhibition constant (Ki) and dissociation constant (Kd) values for binding of POL with SR-B1 receptor*

| **Tested compounds** | **SR-B 1 ki (nM)** | **SR-B 1 kd (nM)** |
| --- | --- | --- |
| **POL** | 3.03 ± 1.71 ^a^ | 3.6 ± 1.3 ^a^ |
| **SOF** | 24.58 ± 3.7 ^b^ | 14.21 ± 2.3 ^b^ |

* POL: *P. ostreatus* lectin and SOF: Sofosbuvir.

** Different letters indicate the significance at p < 0.05.

Table S8. Inhibitory effect and IC_50_ values of POL on the HCV NS3/NS4A protease enzymes compared to SOF*

| **Concentration (nM)** | **HCV** **NS3/NS4A protease inhibitor (%)** | |
| --- | --- | --- |
|  | **POL** | **SOF** |
| **125** | 80.67 | 80.25 |
| **12.5** | 45.13 | 52.06 |
| **1.25** | 26.53 | 26.74 |
| **0.125** | 3.00 | 4.42 |
| **IC_50_ (nM) ± SEM**** | 10.984 ±1.3 ^a^ | 15.54 ± 3.49 ^a^ |

* POL: *P. ostreatus* lectin and SOF: Sofosbuvir.

**The data are expressed as mean ± SEM (n=3) and different letters indicate the significance at

p < 0.05.

Table S9. Inhibitory effect and IC_50_ values POL on the HBV polymerase enzymes compared to LAM*

| **Concentration**  **(nM)** | **HBV polymerase inhibitor (%)** | |
| --- | --- | --- |
|  | **POL** | **LAM** |
| **125** | 84.80 | 88.69 |
| **12.5** | 65.04 | 66.70 |
| **1.25** | 31.70 | 50.76 |
| **0.125** | 15.69 | 22.19 |
| **IC_50_ (nM) ± SEM**** | 4.22 ± 1.13 ^a^ | 6.52 ± 2.43 ^a^ |

* POL: *P. ostreatus* lectin and LAM: Lamivudine.

**The data are expressed as mean ± SEM (n=3) and different letters indicate the significance at p < 0.05.
